# Temperature-dependent rearrangement of gas molecules in ultramicroporous materials for tunable adsorption of CO_2_ and C_2_H_2_

**DOI:** 10.1038/s41467-023-39319-2

**Published:** 2023-06-24

**Authors:** Zhaoqiang Zhang, Yinlin Chen, Kungang Chai, Chengjun Kang, Shing Bo Peh, He Li, Junyu Ren, Xiansong Shi, Xue Han, Catherine Dejoie, Sarah J. Day, Sihai Yang, Dan Zhao

**Affiliations:** 1 Department of Chemical and Biomolecular Engineering, National University of Singapore, 117585 Singapore, Singapore; 2 Department of Chemistry, The University of Manchester, Manchester, M13 9PL UK; 3 School of Chemistry and Chemical Engineering, Guangxi University, Nanning, 530004 China; 4 College of Chemistry, Beijing Normal University, Beijing, 100875 China; 5 The European Synchrotron Radiation Facility, 71 Avenue des Martyrs, CS40220 Cedex 9, 38043 Grenoble, France; 6 Diamond Light Source, Harwell Science Campus, Oxfordshire, OX11 0DE UK; 7 College of Chemistry and Molecular Engineering, Beijing National Laboratory for Molecular Sciences, Peking University, Beijing, 100871 China

**Keywords:** Chemical engineering, Materials chemistry

## Abstract

The interactions between adsorbed gas molecules within porous metal-organic frameworks are crucial to gas selectivity but remain poorly explored. Here, we report the modulation of packing geometries of CO_2_ and C_2_H_2_ clusters within the ultramicroporous CUK-1 material as a function of temperature. In-situ synchrotron X-ray diffraction reveals a unique temperature-dependent reversal of CO_2_ and C_2_H_2_ adsorption affinities on CUK-1, which is validated by gas sorption and dynamic breakthrough experiments, affording high-purity C_2_H_2_ (99.95%) from the equimolar mixture of C_2_H_2_/CO_2_ via a one-step purification process. At low temperatures (<253 K), CUK-1 preferentially adsorbs CO_2_ with both high selectivity (>10) and capacity (170 cm^3^ g^−1^) owing to the formation of CO_2_ tetramers that simultaneously maximize the guest-guest and host-guest interactions. At room temperature, conventionally selective adsorption of C_2_H_2_ is observed. The selectivity reversal, structural robustness, and facile regeneration of CUK-1 suggest its potential for producing high-purity C_2_H_2_ by temperature-swing sorption.

## Abstract (web summary)

Guest clusters within confined nanospaces have a significant impact on molecular recognition. Here authors highlight the potential to systematically control gas-cluster rearrangement, leading to tunable sorption and separation behaviour in a MOF.

## Introduction

Host-guest chemistry is fundamental to the selectivity of many molecular recognition systems^1–5^. The optimization of cooperative interactions, such as electrostatic interactions and hydrogen bonding, plays a crucial role in the design of efficient molecular recognition systems, particularly in porous materials. These cooperative interactions are essential for achieving high performance in gas adsorption, sensing, and catalysis applications^1,3,6–17^. On the other hand, guest-guest interactions or the formation of guest clusters also play an important role in molecular recognition. However, the direct observation and control of guest-guest interactions within confined nanovoids of porous materials is highly challenging and remains poorly explored^18–20^. Screening new host-guest and guest-guest interactions can promote the design of new functional porous materials^1,21^.

Ultramicroporous metal-organic frameworks (MOFs), featuring highly inerratic porosity, tunable pore chemistry, and designable structures, provide a unique platform to explore host-guest interactions^11,22–27^. In particular, the modular nature and reticular structure endow ultramicroporous MOFs with the possibility to precisely control the host-guest and guest-guest interactions within the pores^28,29^. Great advances in host-guest chemistry have been achieved in ultramicroporous MOFs with tailor-made properties for gas adsorption and separation, owing to the confinement effect from the strong host-guest interactions^30–32^. Currently, the major interest in gas adsorption and separation using ultramicroporous MOFs is focused on enhancing recognition selectivity by tuning the host-guest interactions^19,29,33–36^. This is pronounced for selective adsorption of acetylene (C_2_H_2_) from carbon dioxide (CO_2_), as C_2_H_2_ is one of the most important industrial precursors, and the CO_2_ contaminant would be coproduced during the production of C_2_H_2_ via partial combustion of natural gas^34–36^. However, the understanding of the impact of guest-guest interactions or guest clusters on selectivity remains lacking due to the difficulties in the direct observation of such dynamic and weak interactions.

**Fig. 1:**
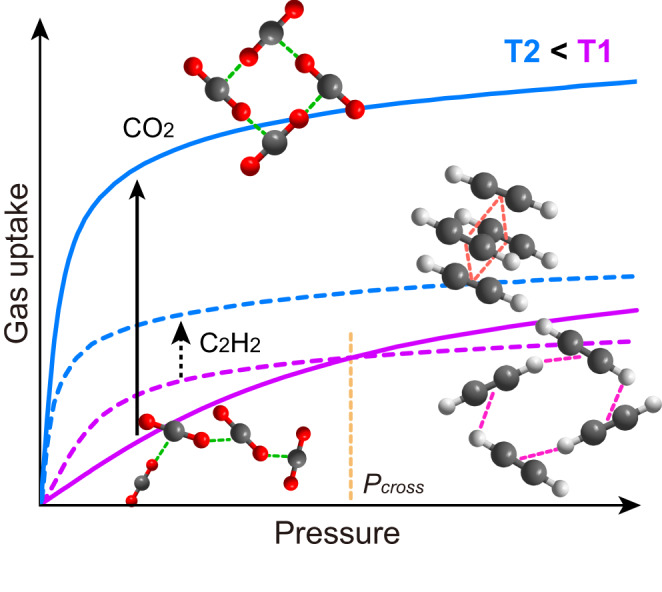
Illustration of the temperature-dependent packing geometries of guest clusters for the normal and inverse adsorption behavior. At a high temperature (T1, purple), the strong host-guest and guest-guest interactions result in preferential adsorption of C_2_H_2_, but the efficient packing of molecular chains formed by CO_2_ molecules through strong guest-guest interactions leads to the higher uptake at the high-pressure range (>*P*_cross_). After decreasing the temperature to T2 (blue), the CO_2_ clusters with T-shaped dimers exhibit higher occupancy of the pore channels than that of C_2_H_2_ clusters packed together via π⋯π interactions, coupled with the strong host-guest interactions, leading to the inverse CO_2_ preferential sorption.

Herein, we report the modulation of geometries of guest-clusters as a function of temperature (Fig. 1) for the normal and inverse selectively and separation of CO_2_ and C_2_H_2_ within the robust ultramicroporous M-CUK-1 (M = Co, Ni, and Mg) materials. The guest-guest interactions and binding domains within CUK-1 with different metal nodes have been observed by in-situ synchrotron X-ray diffractions and molecular simulations. The efficient packing of well-organized CO_2_ clusters with T-shaped dimers gives rise to notably higher crystallographic occupancy and capacity of CO_2_ (106 vs. 86 cm^3^ g^−1^ of C_2_H_2_ in Co-CUK-1 at 298 K), while the stronger host-guest interactions between C_2_H_2_ and CUK-1 at room temperature lead CUK-1 to preferentially adsorb C_2_H_2_ over CO_2_ (Fig. 1). Notably, a much larger increment of CO_2_ capacities at low temperatures was observed compared with those of C_2_H_2_, which is resulted from the highly efficient packing of CO_2_ clusters with tetramers and the significantly stronger host-guest interactions between CO_2_ and CUK-1. This finally leads to much higher CO_2_ capacities (170 vs. 119 cm^3^ g^−1^ of C_2_H_2_ at 233 K) and clear sorption inversion of CO_2_ over C_2_H_2_. Such an inverse CO_2_/C_2_H_2_ adsorption behavior is more desirable for industrial production of C_2_H_2_ via a one-step CO_2_ adsorption process but is rarely investigated^18,29,37–40^. The temperature-dependent reversal of sorption behavior for CO_2_ and C_2_H_2_ is demonstrated by gas sorption isotherms and dynamic breakthrough experiments at various temperatures. High-purity C_2_H_2_ (99.995%) can be directly obtained in a one-step process, and the low energy input for the regeneration suggests that CUK-1 is a promising adsorbent for C_2_H_2_ production via the temperature-swing adsorption (TSA) process.

## Results

### Materials and characterization

M-CUK-1 (M = Co, Ni, and Mg) were hydrothermally synthesized by reacting 2,4-pyridinedicarboxylic acid (2,4-H_2_pdc) and M^2+^-containing salts (M = Co, Ni, and Mg) with KOH in water at 210 °C for 24 h^41–43^. The CUK-1 materials are isostructural. The edge- and vertex-sharing M_3_(µ_3_-OH)_2_ chains serve as undulating pillars connecting the 2,4-pdc ligands in an orthogonal fashion, forming a ‘wine-rack’ topology with one-dimensional diamond-shaped and corrugated channels (Supplementary Fig. 1)^41–43^. All three CUK-1 materials show excellent chemical and structural stability, and are entirely stable upon air exposure for two years (Supplementary Figs. 2–5). Desolvated CUK-1 exhibits an ultramicroporous structure, as evidenced by the negligible N_2_ uptakes and typical type-I CO_2_ isotherms at 77 K and 196 K, respectively (Supplementary Figs. 6–9). The calculated Brunauer–Emmett–Teller (BET) surface areas are 500~600 m^2^ g^−1^ based on the CO_2_ isotherms. Upon desolvation, the exposed μ_3_-OH groups reside orderly in the channels (8.1 × 10.6 Å^2^, Supplementary Fig. 1), acting as potential binding sites to guest molecules through electrostatic interactions^41–43^. This is highly desirable for the adsorption and separation of hydrocarbons.

### Analysis of gas adsorption isotherms and selectivity

**Fig. 2:**
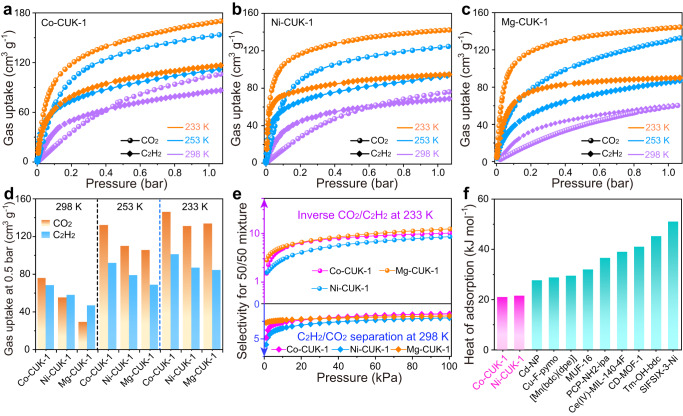
CO2 and C2H2 adsorption and separation performance on CUK-1 materials. The CO_2_ and C_2_H_2_ adsorption isotherms on desolvated Co-CUK-1 (**a**), Ni-CUK-1 (**b**), and Mg-CUK-1 (**c**) at 298 (purple), 253 (blue), and 233 K (orange). **d** The comparison of CO_2_ and C_2_H_2_ uptakes at 0.5 bar on CUK-1 materials at different temperatures. **e** The inverse CO_2_/C_2_H_2_ (1/1) (top) and normal C_2_H_2_/CO_2_ (1/1) (bottom) selectivities at 233 and 298 K, respectively, on CUK-1 materials. **f** Comparison of the zero-coverage heat of adsorption of CUK-1 materials for CO_2_ with those of other materials for inverse CO_2_/C_2_H_2_ separation.

Adsorption isotherms of CO_2_ and C_2_H_2_ on desolvated M-CUK-1 (M = Co, Ni, and Mg) at 298 K indicate the preferential adsorption of C_2_H_2_ at low pressure but higher saturation capacity of CO_2_ upon increasing the pressure (Fig. 2a–c). This behavior results in the intersection of the two isotherms at moderate pressure. Another ultramicroporous compound, SIFSIX-3-Ni, exhibits similar isotherm crossing but with a stronger affinity to CO_2_ at low pressures^29^. After the intersection of CO_2_ and C_2_H_2_ isotherms at 0.42 bar on Co-CUK-1, the CO_2_ isotherm is above that of C_2_H_2_, and CO_2_ uptake at 1 bar can reach 106 cm^3^ g^−1^, much higher than that of C_2_H_2_ (86 cm^3^ g^−1^, Fig. 2a). To the best of our knowledge, such an intersection between CO_2_ and C_2_H_2_ isotherms is rarely observed on porous materials^29^. Similarly, Ni- and Mg-CUK-1 show stronger sorption affinities to C_2_H_2_ in the low-pressure range, and the CO_2_ and C_2_H_2_ isotherms also intersect but with relatively higher intersecting pressures of 0.6 and 0.95 bar on Ni- and Mg-CUK-1, respectively (Fig. 2b, c).

Considering that the inversed CO_2_/C_2_H_2_ selectivity is more desirable for industrial C_2_H_2_ production, the respective guest loadings were measured at progressively lower temperatures to decrease the intersecting pressures. Upon reducing the temperature, there are significant enhancements for CO_2_ uptakes but only a slight increase in C_2_H_2_ uptakes, finally leading to much higher CO_2_ uptakes, even at very low pressures (Fig. 2a–c and Supplementary Figs. 7–9). Specifically, the CO_2_ uptakes at 233 K are 170, 142, and 144 cm^3^ g^−1^ on Co-, Ni-, and Mg-CUK-1 (ca. 4.15, 3.43, and 3.50 CO_2_ molecules per cell, respectively), which notably exceed those of C_2_H_2_ (119, 97, and 89 cm^3^ g^−1^, respectively; ca. 2.89, 2.29, and 2.32 C_2_H_2_ molecules per cell on Co-, Ni-, and Mg-CUK-1, respectively). The densities of adsorbed CO_2_ molecules (based on the structural pore volume) in Co-, Ni-, and Mg-CUK-1 at 233 K were estimated to be 1.40, 1.27, and 1.25 g cm^−3^, respectively. Notably, these densities are higher than that of liquid CO_2_ (1.1 g cm^−3^) but lower than that of dry ice (1.55 to 1.7 g cm^−3^)^44^, indicating the highly efficient packing of CO_2_ in the pores. However, the densities of adsorbed C_2_H_2_ molecules were recorded as only 0.56, 0.50, and 0.45 g cm^−3^ in Co-, Ni-, and Mg-CUK-1, respectively, lower than that of liquid C_2_H_2_ (0.69 g cm^−3^)^45^. At 253 K and 233 K, there is a clear inversion in the adsorption selectivity from C_2_H_2_ to CO_2_ on the CUK-1 materials. The uptake gap between CO_2_ and C_2_H_2_ at 0.5 bar on Co-CUK-1 can reach 40 and 45 cm^3^ g^−1^ at 253 and 233 K (Fig. 2d), respectively.

State-of-the-art C_2_H_2_/CO_2_ separation is mainly realized by cryogenic distillation and solvent absorption with high energy penalty. The adsorptive separation using CO_2_-selective other than C_2_H_2_-selective materials is preferable in the industry for producing pure C_2_H_2_ via one-step sorption procedures. To see whether such a temperature-induced adsorption inversion behavior can be used for inverse CO_2_/C_2_H_2_ separation, we evaluated CUK-1 materials for separating the equimolar mixture of CO_2_/C_2_H_2_ by analyzing the single-component isotherms via ideal adsorbed solution theory (IAST). At 298 K, CUK-1 only shows a moderate C_2_H_2_/CO_2_ selectivity of ca. 2. However, at 233 K, CUK-1 exhibits the inversed CO_2_/C_2_H_2_ selectivity of 9.5, 8.4, and 12.1 for Co-, Ni-, and Mg-CUK-1, respectively (Fig. 2e). The inversed selectivities are comparable with those of the state-of-the-art materials for inversed CO_2_/C_2_H_2_ separation, such as [Mn(bdc)(bpe)] (9)^46^, Ce(IV)-MIL-140-4F (9.6)^37^, PCP-NH_2_-ipa (6.4)^35^, and SIFSIX-3-Ni (7.5)^29^, but lower than the benchmark material Cu-F-pymo (> 10^5^)^38^. Furthermore, the isosteric heats of adsorption (*ΔH*_*ads*_) of CO_2_ on Co- and Ni-CUK-1 were calculated to be 20.8 and 21.7 kJ mol^−1^, respectively (Supplementary Fig. 10), much lower than that of other materials (Fig. 2f), such as PCP-NH_2_-ipa (26.8 kJ mol^−1^)^35^, [Mn(bdc)(bpe)] (29.5 kJ mol^−1^)^46^, MUF-16 (32 kJ mol^−1^)^39^, and Tm_2_(OH-bdc) (45 kJ mol^−1^)^40^.

### Guest configurations determined by in-situ synchrotron X-ray powder diffraction

**Fig. 3:**
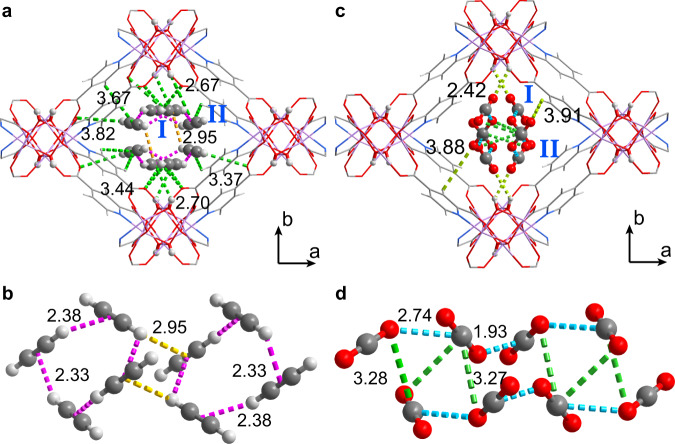
Configurations of adsorbed C2H2 and CO2 molecules within Co-CUK-1 from refinements of in-situ synchrotron X-ray powder diffraction data at 298 K. Views of host-guest interactions of C_2_H_2_ (**a**) and CO_2_ (**c**) in Co-CUK-1. Packing geometries of C_2_H_2_ (**b**) and CO_2_ (**d**) clusters. Color code: C, gray; H, gray-25%; O, red; N, blue; Co, pink.

**Fig. 4:**
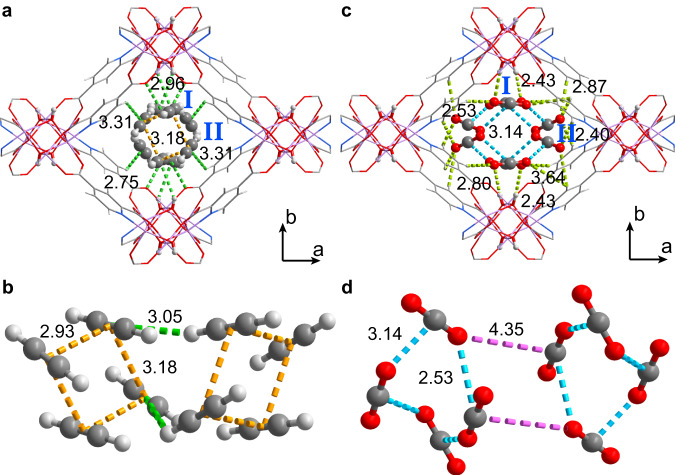
Configurations of adsorbed C2H2 and CO2 molecules within Co-CUK-1 from refinements of in-situ synchrotron X-ray powder diffractions at 233 K. Views of host-guest interactions between C_2_H_2_ (**a**) and CO_2_ (**c**) in Co-CUK-1. Packing geometries of C_2_H_2_ (**b**) and CO_2_ (**d**) clusters. Color code: C, gray; H, gray-25%; O, red; N, blue; Co, pink.

In-situ synchrotron X-ray powder diffraction data on CO_2_- and C_2_H_2_-loaded CUK-1 materials were collected as a function of temperature (Supplementary Figs. 11–12). Full refinements of the data indicate two binding sites in the asymmetric unit: site I is close to the μ_3_-OH group, and site II locates near the pore surface (Figs. 3–4 and Supplementary Figs. 13–18). At 298 K, the total crystallographic occupancy of C_2_H_2_ molecules (2.05 per cell) in Co-CUK-1 is in excellent agreement with that obtained from the isotherm (2.07 C_2_H_2_ per cell). C_2_H_2_ molecules at site I locate almost perpendicular to μ_3_-OH groups, forming O-H⋯π_C2H2_ H-bonds (2.92 Å, dotted green lines), supplemented by additional interactions via C-H_C2H2_⋯O_ligand_ H-bonding (dotted green lines, 2.67-2.71 Å, Fig. 3a and Supplementary Fig. 13a). C_2_H_2_ molecules at site II sit close to the aromatic rings on the pore surface and form weak interactions with the framework through multiple C-H_C2H2_⋯O_ligand_ H-bonding (3.37–3.82 Å) and π_C2H2_⋯H_ligand_ (3.44–3.67 Å) interactions. Moreover, at high loading, the neighboring C_2_H_2_ molecules synergistically interact with each other through multiple H_C2H2_⋯π_C2H2_ interactions (dotted pink lines, 2.33–2.95 Å), forming the tetramer-clusters of C_2_H_2_ (Fig. 3b).

In contrast, CO_2_ molecules show different geometries of packing (Fig. 3c and Supplementary Fig. 14a). CO_2_ molecules at site I exhibit an end-on interaction to μ_3_-OH group via hydrogen bonds (dotted lime lines, 2.42 Å of O-H⋯O_CO2_), but no interactions between CO_2_ and the ligand of CUK-1 were observed. CO_2_ molecules at site II interact with the pore surface via weak O⋯H_ligand_ interactions (3.88–3.91 Å, Supplementary Fig. 14a). Thus, adsorbed CO_2_ molecules at both sites show much weaker interactions compared with C_2_H_2_, entirely consistent with the adsorption results at room temperature. However, at high loading, two one-dimensional chains of CO_2_ (dotted azure lines) running along the channel were formed via strong guest-guest interactions (2.93 and 2.74 Å, Fig. 3d). These chains are stabilized by multiple weak intermolecular dipole interactions between monomer-to-dimer and dimer-to-dimer of CO_2_. Furthermore, two chains interact with each other via multiple synergistic host-host interactions (dotted green lines, 3.27–3.28 Å). Notably, the neighboring CO_2_ molecules exhibit a head-to-center (C = O⋯C) geometry (Supplementary Fig. 14a), thus leading to the efficient packing of CO_2_ in the pore channels. Similar binding sites of CO_2_ in Ni-CUK-1 were also observed (Supplementary Fig. 18). Compared with C_2_H_2_ clusters, the efficient packing of CO_2_ molecules near the center of pore channels via strong guest-guest interactions but with less host-guest interactions is the main reason for the high adsorption of CO_2_ in CUK-1 at high pressures.

At 233 K, remarkable changes in the packing geometry of CO_2_ and C_2_H_2_ were observed (Fig. 4), and the crystallographic occupancy of C_2_H_2_ molecules increased to 2.77 per cell. Meanwhile, the CO_2_ occupancy increased to 3.47 per cell (vs. 2.07 at 298 K), indicating the high capacity of Co-CUK-1 for CO_2_ at 233 K compared with that for C_2_H_2_. This is entirely consistent with the isotherms. C_2_H_2_ molecules at site I interact with bridging μ_3_-OH groups via π_C2H2_⋯H-O H-bond (2.96 Å) that is supplemented by weak C-H⋯O_ligand_ H-bonding (dotted green lines, 2.75 Å, Fig. 4a and Supplementary Fig. 13b). CO_2_ molecules at site I are stabilized by C-O_CO2_⋯H_μ3-OH_ H-bonding (2.43 Å) and C-O_CO2_⋯H_ligand_ interactions (2.80 and 3.64 Å, Fig. 4c and Supplementary Fig. 14b). C_2_H_2_ molecules at site II reside near the center of pore channels with fewer host-guest interactions (π⋯H_ligand_ 3.31 Å), similar to that of CO_2_ in the channels at 298 K. However, CO_2_ molecules are located in the corner of the pore channels and stabilized by multiple weak host-guest interactions (C-O_CO2_⋯H_ligand_, 2.40–2.87 Å), thus leading to the strong binding affinities of host framework for CO_2_. Meanwhile, in Co-CUK-1, C_2_H_2_ clusters are formed with C_2_H_2_ molecules via π_C2H2_⋯π_C2H2_ interactions (dotted orange lines, 2.95 and 3.18 Å, Fig. 4b). The neighboring clusters synergistically interact with each other via weak C-H⋯π H-bonding (3.05 and 3.17 Å), leading to the efficient packing of C_2_H_2_ molecules. By contrast, the isolated CO_2_ clusters are formed with four CO_2_ molecules by closely interacting with each other (distances of 2.53 and 3.14 Å) with the head-to-center configurations, forming the quasi-T-shaped geometry (C = O⋯C, dotted azure lines, Fig. 4d). This is similar to that in dry ice, indicating the highly efficient packing of CO_2_ molecules (thus packing densities) in Co-CUK-1. Similar CO_2_ clusters with quasi-T-shaped dimers were also observed in Ni-CUK-1 at 233 K (Supplementary Fig. 16d). Thus, the notably stronger guest-guest interactions between adsorbed CO_2_ molecules at low temperature promote the unusually selective adsorption of CO_2_ over C_2_H_2_ at 233 K. Importantly, to the best of our knowledge, such guest-guest packing geometries and host-guest interactions at different temperatures have not been observed in porous materials yet.

To quantitatively compare the binding affinities of CUK-1 to CO_2_ and C_2_H_2_ at different temperatures, the static binding energies (*ΔE*) were further estimated by first-principles density functional theory (DFT) calculations (Supplementary Tables 2 and 3). The results show that after decreasing the temperature from 298 to 233 K, there are significant increases of *ΔE* at site I for CO_2_. Especially, *ΔE* for CO_2_ at site I on Co-CUK-1 is 43.5 kJ mol^−1^, much higher than that for C_2_H_2_ (33.3 kJ mol^−1^), directly validating the preferential adsorption of CO_2_ over C_2_H_2_. There is a subtle difference in host-guest interactions when varying the metal nodes in CUK-1 (Supplementary Tables 2 and 3), and this has little influence on the formation of guest clusters and the tunable CO_2_ and C_2_H_2_ sorption behavior. Thus, the guest-guest interactions and/or the arrangement of guest clusters play the dominant role in the inverse CO_2_ sorption of CUK-1 materials.

### Dynamic breakthrough tests

**Fig. 5:**
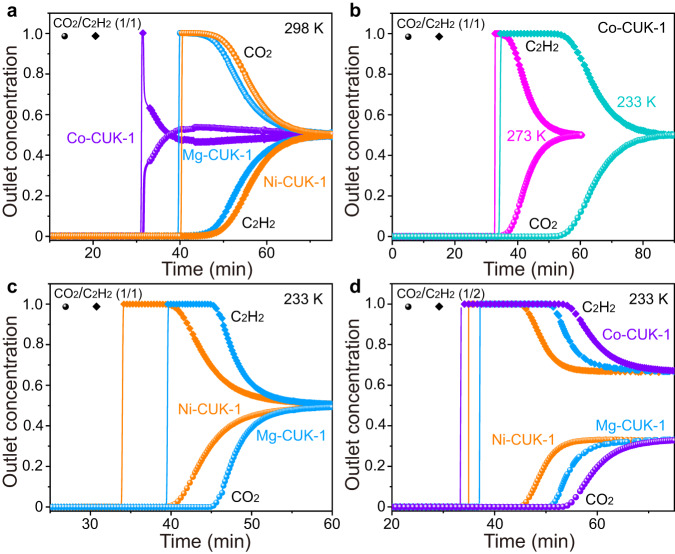
Breakthrough curves of CUK-1 materials for CO2/C2H2 mixtures. **a** Breakthrough curves of CO_2_/C_2_H_2_ (1/1) mixture on CUK-1 materials at 298 K with a flow rate of 2.0 mL min^−1^. **b** Breakthrough curves of CO_2_/C_2_H_2_ (1/1) mixture on Co-CUK-1 at 273 and 233 K with flow rates of 2.0 and 3.0 mL min^−1^, respectively. **c** Breakthrough curves of CO_2_/C_2_H_2_ (1/1) mixture on Ni-CUK-1 and Mg-CUK-1 at 233 K with a flow rate of 3.0 mL min^−1^. **d** Breakthrough curves of CO_2_/C_2_H_2_ (1/2) mixture on CUK-1 materials at 233 K with a flow rate of 3.0 mL min^−1^.

Dynamic breakthrough experiments on CUK-1 materials using mixtures of CO_2_/C_2_H_2_ were conducted (Fig. 5). For the equimolar CO_2_/C_2_H_2_ mixture at 298 K, Ni- and Mg-CUK-1 show typical C_2_H_2_-preferential sorption over CO_2_ with a clear separation of C_2_H_2_ and CO_2_, but Co-CUK-1 shows very poor separation (Fig. 5a and Supplementary Fig. 19). These are consistent with the isotherm results at 298 K. At 273 K, a clear inversed CO_2_/C_2_H_2_ separation was observed on Co-CUK-1 (Fig. 5b and Supplementary Fig. 20), and there is an obvious deterioration in C_2_H_2_/CO_2_ separation performance on Ni- and Mg-CUK-1 (Supplementary Fig. 21). At 233 K, an evident inversed CO_2_/C_2_H_2_ separation was observed on CUK-1 materials, and all materials exhibit highly selective adsorption of CO_2_ over C_2_H_2_ (Fig. 5b, c). The dynamic CO_2_ uptake capacities at 233 K were calculated to be 140, 110, and 122 cm^3^ g^−1^ on Co-, Ni-, and Mg-CUK-1, respectively, much higher than those of C_2_H_2_ (62, 67, and 78 cm^3^ g^−1^, respectively). To mimic the industrial processes for C_2_H_2_ production, we further studied a gas mixture of CO_2_/C_2_H_2_ (1/2). A complete inversed CO_2_/C_2_H_2_ separation was realized with CO_2_ retained in the fixed bed for a longer duration (Fig. 5d). Significantly, the productivity of pure C_2_H_2_ (99.995%) can reach 62 and 41.7 L kg^−1^ on Co-CUK-1 and Mg-CUK-1, respectively, much higher than that on MUF-16 (27 L kg^−1^)^39^ and Cu-F-pymo (3.7 L kg^−1^)^38^. It is worth noting that these materials show excellent cycling separation performance and can be easily regenerated by purging helium (He) at 298 K (Supplementary Figs. 22–23). The notably high CO_2_ uptake and facile regeneration of CUK-1 are particularly desirable for practical applications to reduce the energy footprint compared with state-of-the-art cryogenic distillations.

## Discussion

In summary, we report the direct observation of packing geometry rearrangement of gas clusters as a function of temperature to control the adsorption selectivity of CO_2_ and C_2_H_2_ on ultramicroporous MOFs. The strong host-guest interactions of CUK-1 for C_2_H_2_ at ambient conditions led to the preferential adsorption of C_2_H_2_. However, the efficient packing of CO_2_ molecules via strong guest-guest interactions forms CO_2_ clusters, leading to higher CO_2_ capacity. Impressively, after decreasing temperature, the host-guest interactions between CO_2_ and host framework became stronger than that for C_2_H_2_. Furthermore, the highly ordered arrangement of CO_2_ clusters with the T-shaped dimers endows CUK-1 with remarkably higher capacities for CO_2_ over those for C_2_H_2_. Such host-guest interactions, guest-guest interactions, and gas cluster formation were elucidated by in-situ synchrotron X-ray powder diffraction and molecular simulations. This idiosyncratic inversion of the adsorption behavior of C_2_H_2_ and CO_2_ was verified by dynamic breakthrough experiments with high-purity C_2_H_2_ (99.995%) obtained in a one-step process. Furthermore, the fixed-bed packed with CUK-1 can be easily regenerated at room temperature by purging an inert gas, indicating that the TSA process using CUK-1 materials is highly efficient for C_2_H_2_ production.

## Methods

### Gas adsorption and separation experiments

CO_2_ and C_2_H_2_ sorption isotherms were collected at different temperatures on a Micromeritics ASAP 2020 instrument equipped with commercial software for data calculation and analysis. The test temperatures were controlled by soaking the sample cell in a circulating water bath (298 K), ice/methanol mixture (233–273 K), dry ice/acetone mixtures (196 K), or liquid N_2_ (77 K). Before measurement, the sample (80–100 mg) was degassed at 423 K for 24 h. The breakthrough experiments were performed in a stainless-steel fixed bed (4.6 mm inner diameter × 50 mm length) packed with ~0.6 g of CUK-1 powder. Before the breakthrough experiment, the fixed bed was heated at 423 K under a flow of He for complete activation. The fixed bed was then cooled to room temperature and immersed in a water/methanol bath with different temperatures. Then, the gas mixtures (C_2_H_2_/CO_2_) were introduced, and the outlet gas was monitored by mass spectrometry (Hidden QGA quantitative gas analysis system).

### In-situ synchrotron powder X-ray diffraction and structure determination

Fresh samples of Co-CUK-1 or Ni-CUK-1 were pre-activated under a dynamic vacuum at 200 °C, then loaded into a 0.7 mm borosilicate capillary under an inert atmosphere. Then the capillary was further activated by heating to 100 °C under a dynamic vacuum for 2 h before cooling down to room temperature. For samples measured under 233 K, synchrotron X-ray powder diffraction was carried out on the ID22 high-resolution powder diffraction beamline at the European Synchrotron Radiation Facility (ESRF). C_2_H_2_ or CO_2_ was introduced into the capillary, and diffraction data were collected after one-hour stabilization. Data were measured between 0 and 35° with a 13-channel multi-analyzer stage under the wavelength of 0.354267(4) Å. Data were binned using a step size of 0.002°. For samples measured under 298 K, high-resolution powder X-ray diffraction patterns were collected on the powder diffractometer (λ = 0.825829(1) Å) at room temperature on beamline I11 (Diamond Light Source, UK). C_2_H_2_ or CO_2_ was dosed offline and then sealed for measurement. Data were collected between 0 and 150° using a step size of 0.001° with five multi-analyzing crystal (MAC) detectors without further re-binned.

Pawley and Rietveld refinements of the structure were carried out using the TOPAS software package (roughly between 18–0.90 Å in d-spacing). Due to the high flexibility of the framework, index with constraints was used to get the information on cell parameters and space groups. Stephen fitting was applied to describe the diffraction peaks and their anisotropic broadening. Approximate positions of the guest molecule under a rigid body model were found using the simulated annealing approach before further refinement was used to find the optimal orientation of the guest molecules. The final refined structural parameters include cell parameters, the fractional coordinates (*x*, *y*, *z*) and isotropic displacement factors for all atoms except hydrogen, and the site occupancy factors (SOF) for guest molecules. The accuracy of the final model was verified by the convergence of the weighted profile factor (*R*_wp_), the chemical sense of the model, and the good correlation between the observed and calculated diffraction patterns.

## Supplementary information

Supplementary Information

Peer review file

## Data Availability

Crystallographic data for the structures reported in this article have been deposited at the Cambridge Crystallographic Data Centre under deposition numbers CCDC 2214437, 2214440–2214446. These data can be obtained free of charge via https://www.ccdc.cam.ac.uk/structures/. All the other relevant data, additional graphics, and calculations that support the findings of this study are available within the article and its Supplementary Information, or from the corresponding authors upon request.
