# Supplementary material for: Temperature-dependent rearrangement of gas molecules in ultramicroporous materials for tunable adsorption of CO2 and C2H2
Source: Nat Commun. 2023 Jun 24;14:3789. doi: 10.1038/s41467-023-39319-2 (PMC10290667; doi:10.1038/s41467-023-39319-2)
Supplement: Supplementary file 1 — Supplementary Information [file 41467_2023_39319_MOESM1_ESM.pdf]

Supplementary Information

Temperature-dependent rearrangement of gas molecules in ultramicroporous materials for tunable adsorption of CO_2 and C_2H_2

By Zhang et al.

Table of Contents

Supplementary Methods	S3
Supplementary Figures	S5
Supplementary Tables	S18
References	S23

Supplementary Methods

TGA measurements

Thermogravimetric analyses (TGA) were performed on a TA Instruments STD-600 equipment at a heating rate of 10 °C min^{-1} with an N_2 flow rate of 50 mL min^{-1}. The sample holders were alumina crucibles, and the amount of sample used in each measurement was 8 (± 2) mg. The data collected were analyzed using Universal Analysis software (version 4.4A) from TA Instruments.

Powder X-ray diffraction analyses

Powder X-ray diffraction (PXRD) patterns were collected using a Rigaku Miniflex 600 diffractometer (Cu Kα λ = 1.540598 Å) with an operating power of 40 kV, 15 mA, and a scan rate of 2.0 ° min^{-1}. The data were collected in a two-theta range of 2–30°.

IAST selectivity calculations

The pyIAST package[1] was used to perform the IAST calculations and predict the sorption performance of porous materials for binary mixed gas. The isotherm data for CO_2 and C_2H_2 were first fitted with a dual-site Langmuir-Freundlich isotherm model:[2,3]

$$q = q_{A,sat}\frac{b_A p^{\nu_A}}{1 + b_A p^{\nu_A}} + q_{B,sat}\frac{b_B p^{\nu_B}}{1 + b_B p^{\nu_B}} \qquad (1)$$

The adsorption selectivity for $C_2H/_2CO_2$ separation is defined by the following:

$$S_{ads} = \frac{q_1/q_2}{y_1/y_2} \qquad (2)$$

where q_1 and q_2 are the molar loadings in the adsorbed phase in the mixture, mmol g^{-1}; y_1 and y_2 ($y_2 = 1 - y_1$) represent the mole fractions of CO_2 and C_2H_2 in the feed gas.

Isosteric heat of adsorption calculations

The isosteric enthalpy of adsorption, ΔH_{ads}, can be estimated using the *Clausius-Clapeyron* equation:

$$\Delta H_{ads} = RT^2\left(\frac{\partial lnp}{\partial T}\right)_q \qquad (3)$$

where ΔH (kJ mol^{-1}) is the isosteric heat of adsorption at a specific surface loading of adsorbate, R (kJ·mol^{-1}·K^{-1}) is the universal gas constant, T (K) is the temperature, p (kPa) is the pressure, and q (mmol g^{-1}) is the adsorbate amount

adsorbed on the surface.

Integrating the equation can give the following equation:

$$lnp = -\frac{\Delta H_{ads}}{RT} + C \quad (4)$$

where *C* is an integral constant.

DFT calculations

First-principles density functional theory (DFT) calculations were performed in Castep software (*BIOVIA Materials Studio*).[4] A semi-empirical addition of dispersive forces to conventional DFT was included in the calculation to account for van der Waals interactions. Vanderbilt-type ultra-soft pseudopotentials and generalized gradient approximation with Perdew–Burke–Ernzerhof exchange correlation were used. A cutoff energy of 590 eV and a 1 × 1 × 2 *k*-point mesh (generated using the Monkhorst-Pack scheme) were found to be enough for the total energy to converge within 0.01 meV atom^{-1}. To obtain the gas binding energy, the structures of the refined structures from in-situ synchrotron powder X-ray diffraction experiments with gas molecules deleted were first optimized, followed by a full structural relaxation. Then, a gas molecule in a supercell (the refined structures loaded with gas molecules from in-situ synchrotron powder X-ray diffraction experiments) was also relaxed as a reference. The static binding energy (at $T = 0$ K) was then calculated using: $E_B = E_{(MOF)} + E_{(gas)} - E_{(MOF + gas)}$.

Supplementary Figures

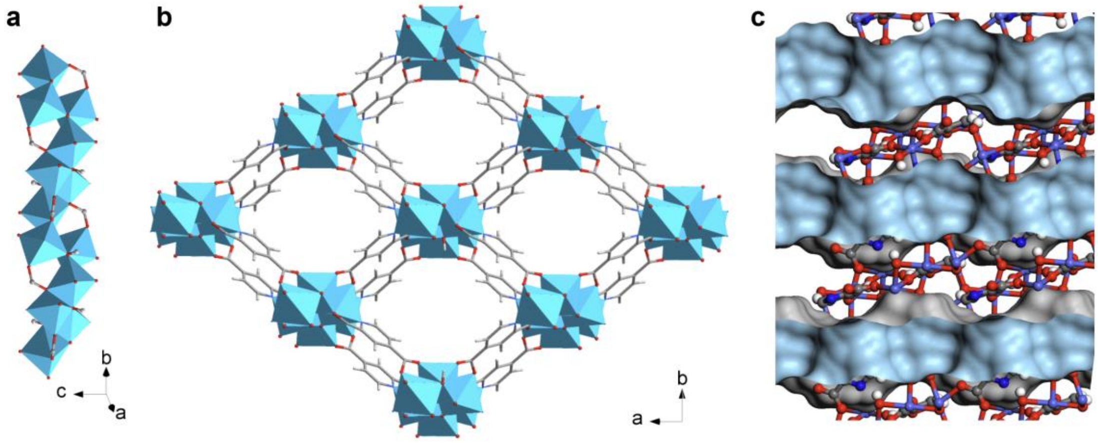

Supplementary Figure 1. (a) View of coordination mode of metal ions in CUK-1; (b) Crystal structure of desolvated CUK-1; (c) Connolly pore surface of the corrugated pore channels in CUK-1 materials.

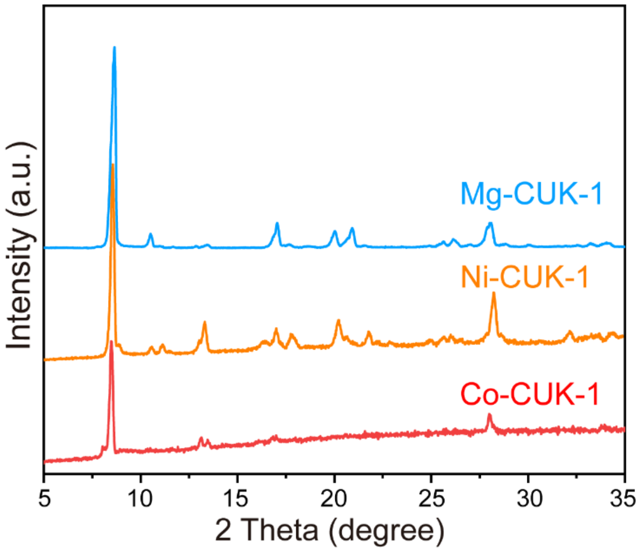

Supplementary Figure 2. PXRD patterns of the as-synthesized CUK-1 materials.

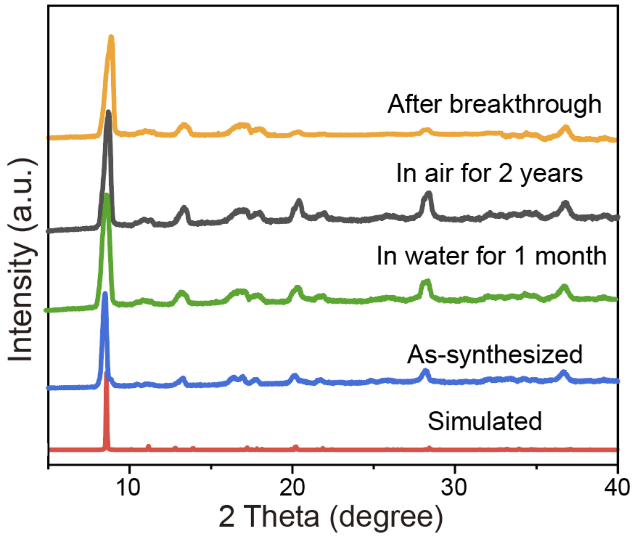

Supplementary Figure 3. PXRD patterns of Ni-CUK-1 under different conditions.

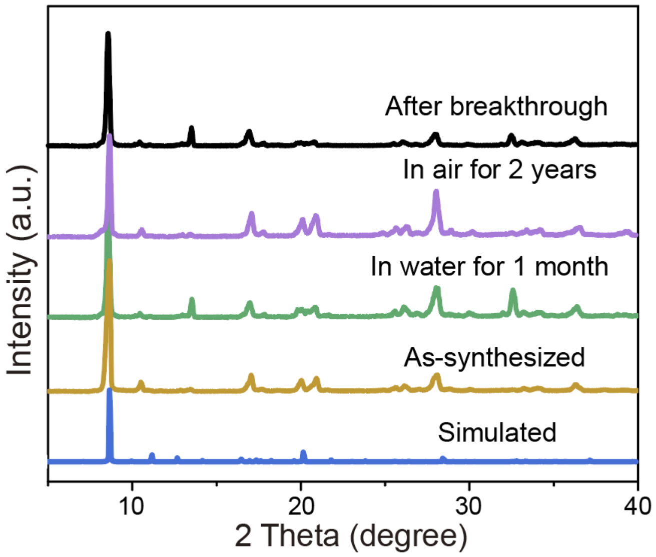

Supplementary Figure 4. PXRD patterns of Mg-CUK-1 under different conditions.

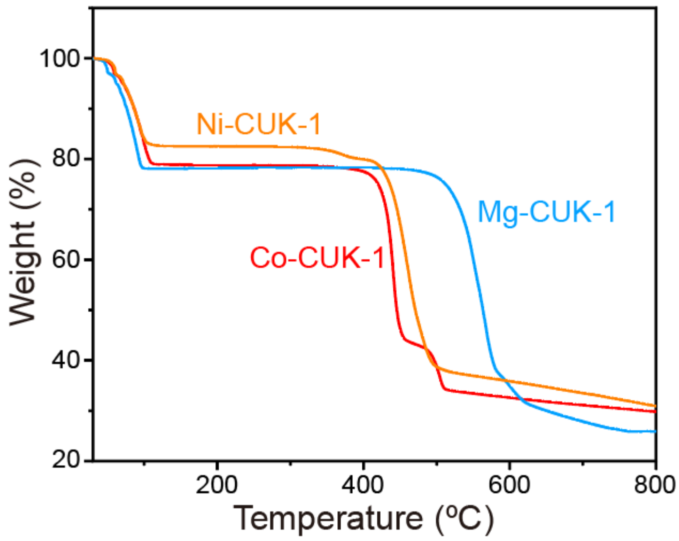

Supplementary Figure 5. TGA curves of CUK-1 materials.

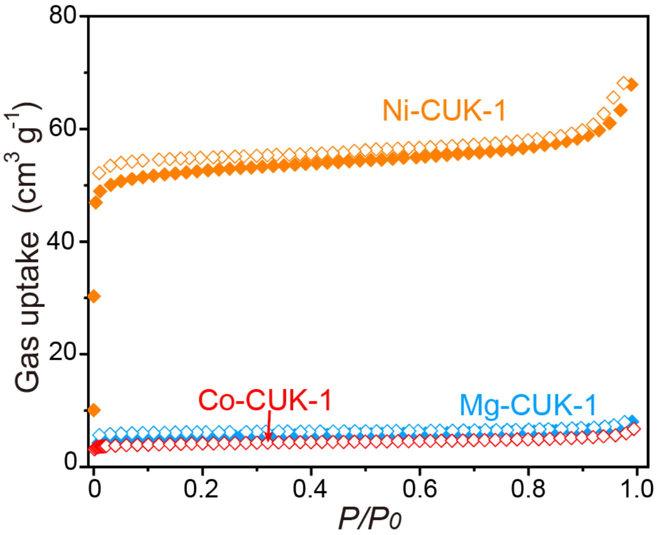

Supplementary Figure 6. N_2 sorption isotherms collected at 77 K on CUK-1 materials (filled, adsorption; open, desorption).

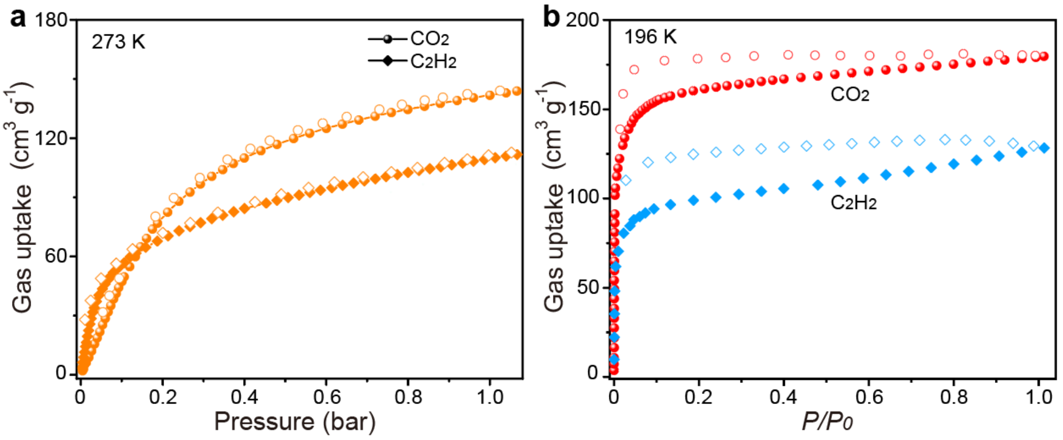

Supplementary Figure 7. The CO_2 and C_2H_2 sorption isotherms on Co-CUK-1 at 273 (a) and 196 K (b). **Note:** The hysteresis at 196 K may be due to the kinetic effect as the pore size is similar to the molecular sizes of CO_2 and C_2H_2 (Co-CUK-1 possesses ultramicropores, as shown in Supplementary Fig. 5).

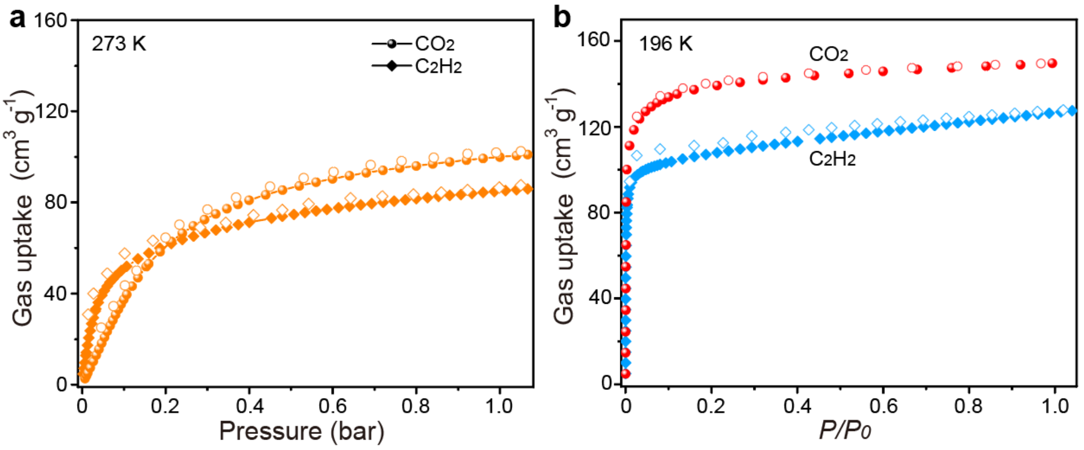

Supplementary Figure 8. The CO_2 and C_2H_2 sorption isotherms on Ni-CUK-1 at 273 (a) and 196 K (b).

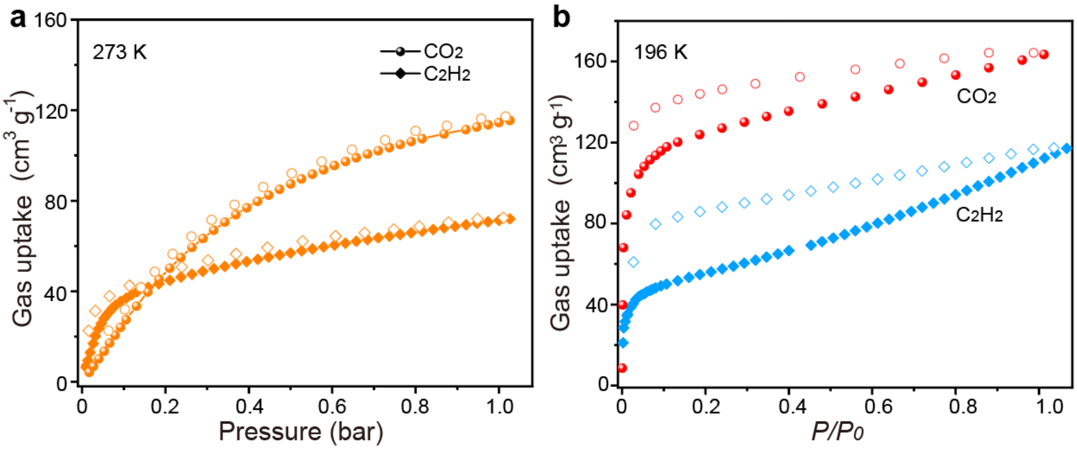

Supplementary Figure 9. The CO_2 and C_2H_2 sorption isotherms on Mg-CUK-1 at 273 (a) and 196 K (b). **Note:** The hysteresis in 196 K data may be due to the kinetic effect as the pore size is similar to the molecular sizes of CO_2 and C_2H_2 (Mg-CUK-1 possesses ultramicropores, as shown in Supplementary Fig. 5).

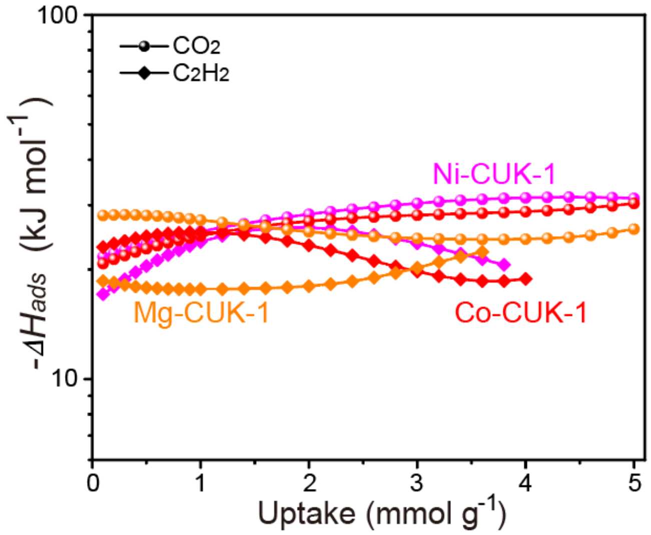

Supplementary Figure 10. Isosteric adsorption enthalpy (ΔH_{ads}) of CO_2 (circle) and C_2H_2 (square) on CUK-1 materials.

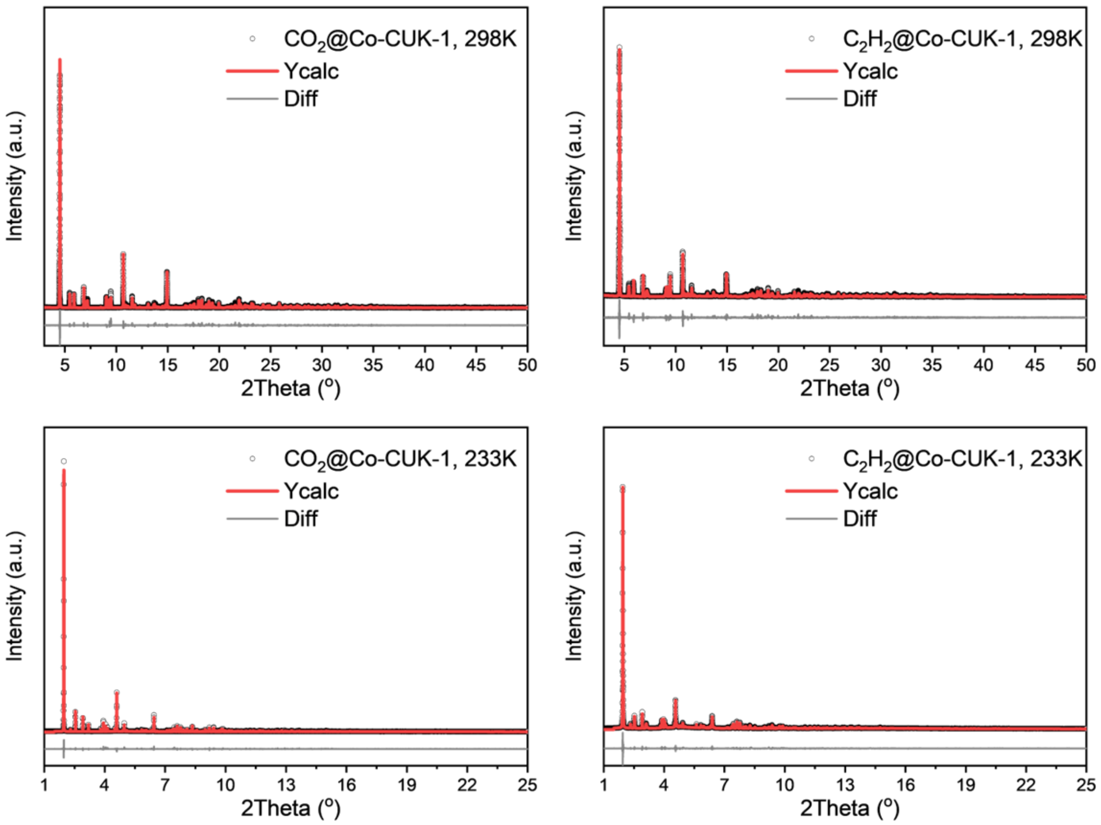

Supplementary Figure 11. Synchrotron X-ray diffraction patterns and Rietveld refinement for guest-loaded Co-CUK-1 under 233 K and 298 K (black circle: raw data; red line: calculated PXRD pattern; gray line: the difference between observed and fitted patterns).

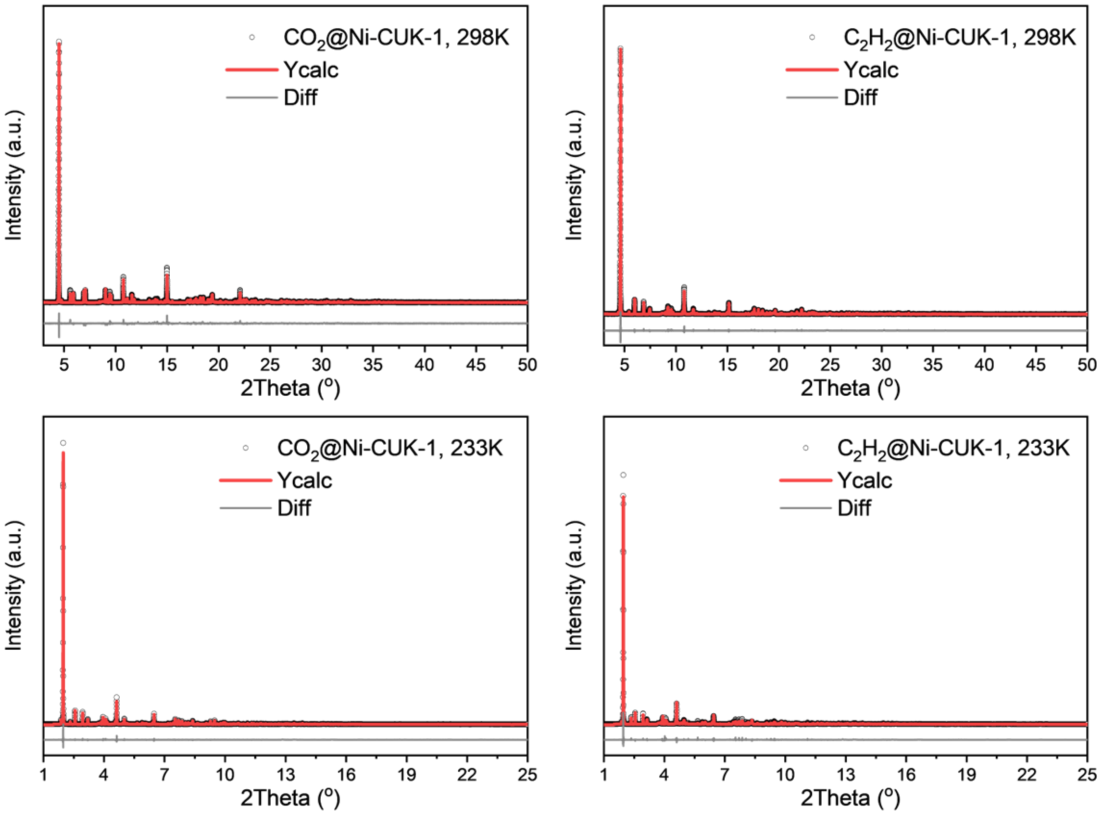

Supplementary Figure 12. Synchrotron X-ray diffraction patterns and Rietveld refinement for guest-loaded Ni-CUK-1 under 233 K and 298 K (black circle: raw data; red line: calculated PXRD pattern; gray line: the difference between observed and fitted patterns).

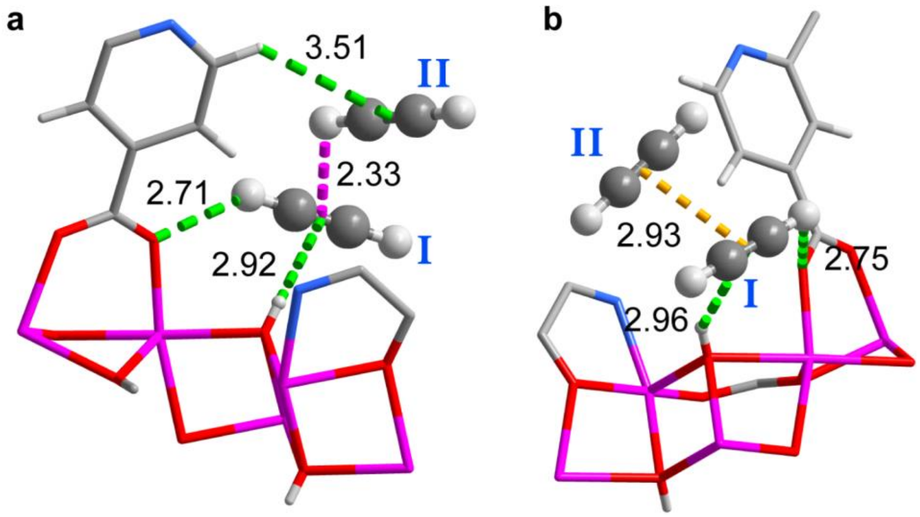

Supplementary Figure 13. Views of C_2H_2 configurations at two binding sites in Co-CUK-1 at 298 (a) and 233 K (b) determined by synchrotron X-ray powder diffraction data. Color code: C, gray; H, gray-25%; O, red; N, blue; Co, pink.

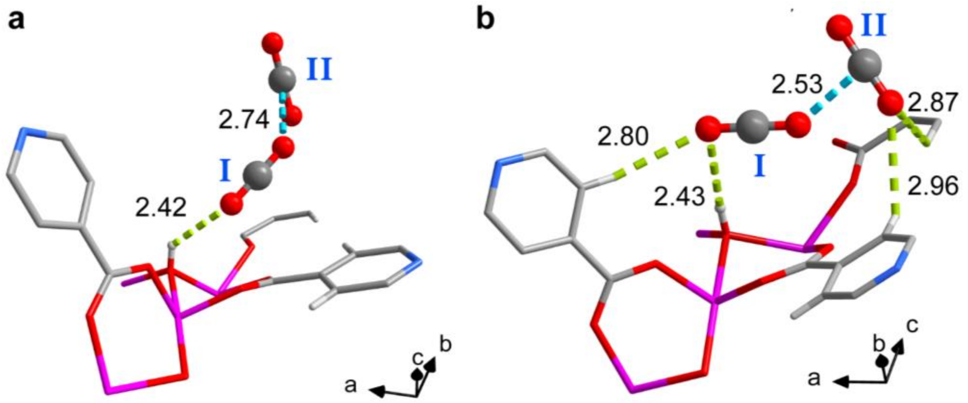

Supplementary Figure 14. Views of CO_2 configurations in Co-CUK-1 at 298 (a) and 233 K (b) determined by synchrotron X-ray powder diffraction data. Color code: C, gray; H, gray-25%; O, red; N, blue; Co, pink.

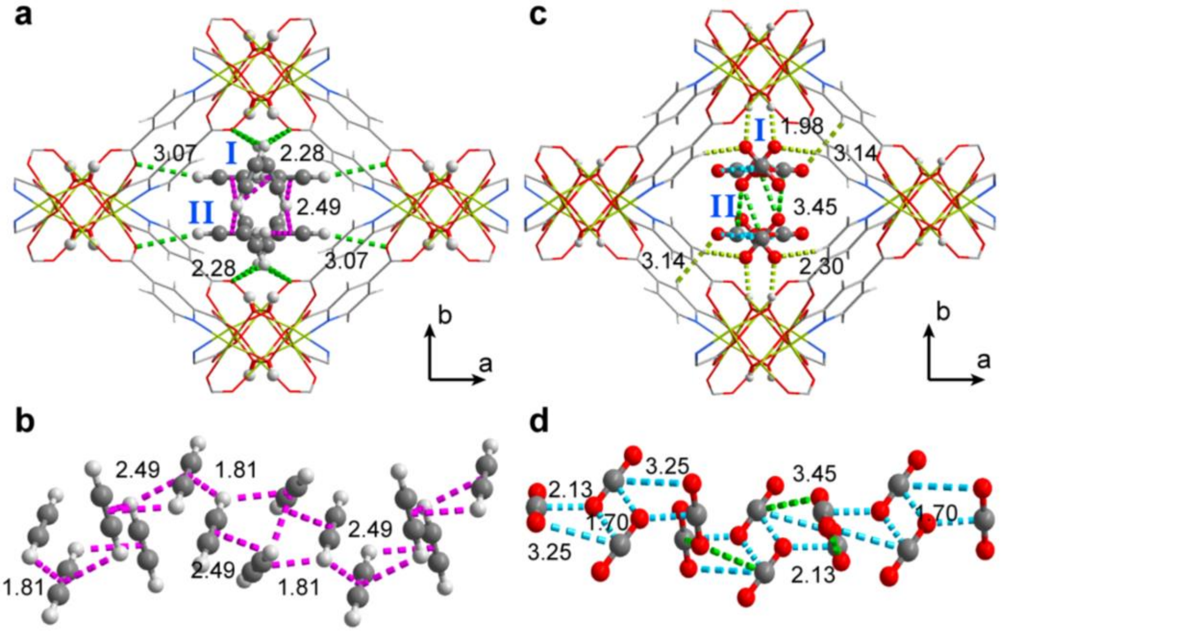

Supplementary Figure 15. Views of C_2H_2 (a and b) and CO_2 (c and d) configurations in Ni-CUK-1 determined by synchrotron X-ray powder diffraction data at 298 K. Color code: C, gray; H, gray-25%; O, red; N, blue; Ni, light green.

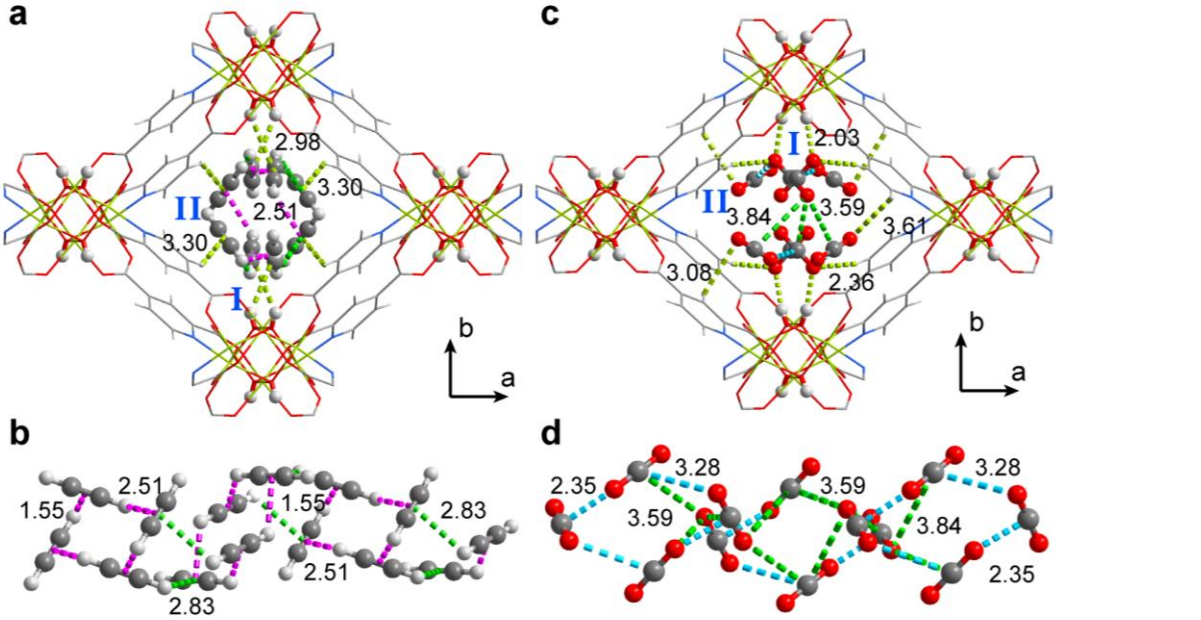

Supplementary Figure 16. Views of C_2H_2 (a and b) and CO_2 (c and d) configurations in Ni-CUK-1 determined by synchrotron X-ray powder diffraction data at 233 K. Color code: C, gray; H, gray-25%; O, red; N, blue; Ni, light green.

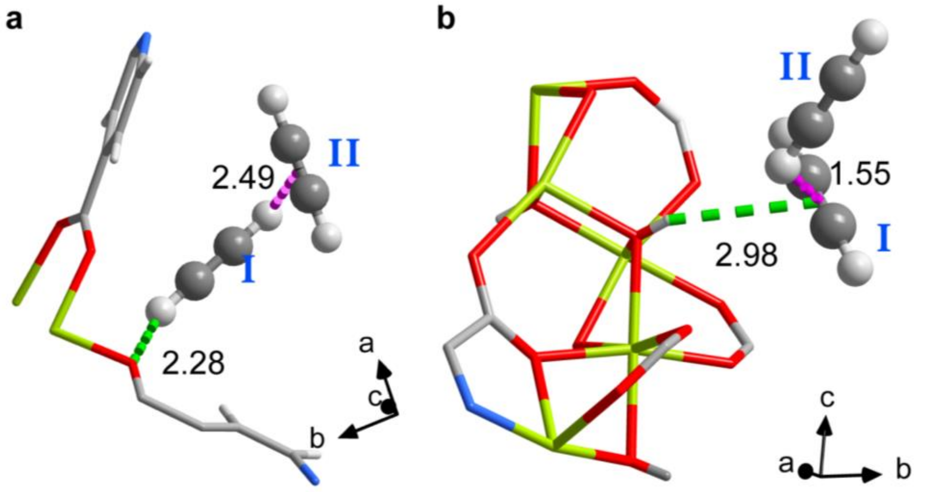

Supplementary Figure 17. Views of C_2H_2 configurations at two binding sites in Ni-CUK-1 at 298 (a) and 233 K (b) determined by synchrotron X-ray powder diffraction data. Color code: C, gray; H, gray-25%; O, red; N, blue; Ni, light green.

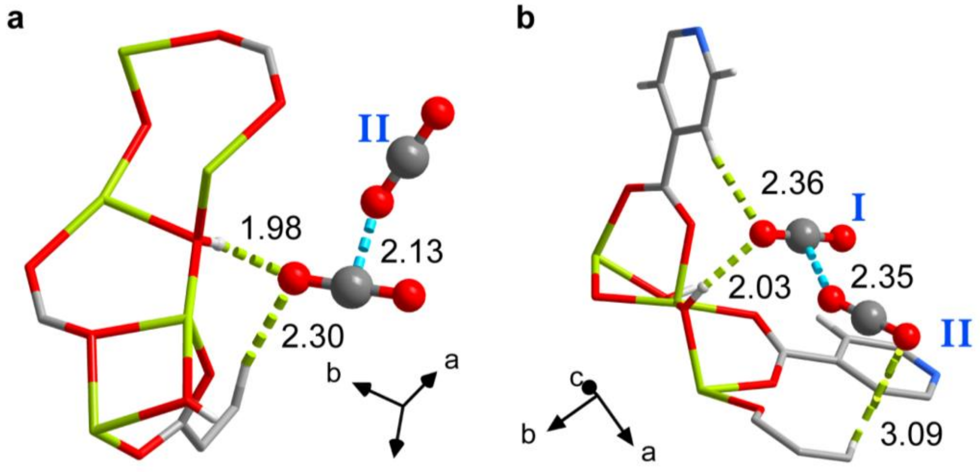

Supplementary Figure 18. Views of CO_2 configurations in Ni-CUK-1 at 298 (a) and 233 K (b) determined by synchrotron X-ray powder diffraction data. Color code: C, gray; H, gray-25%; O, red; N, blue; Ni, light green.

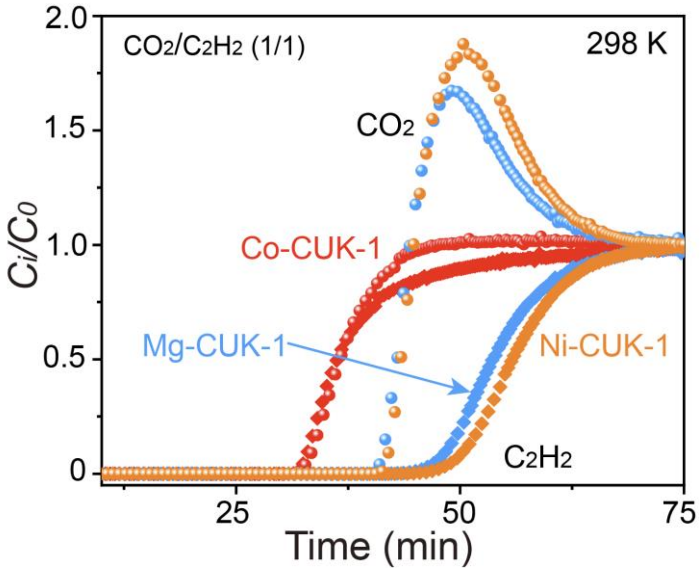

Supplementary Figure 19. Experimental breakthrough curves of CO_2/C_2H_2 (1/1) mixture on CUK-1 materials at 298 K.

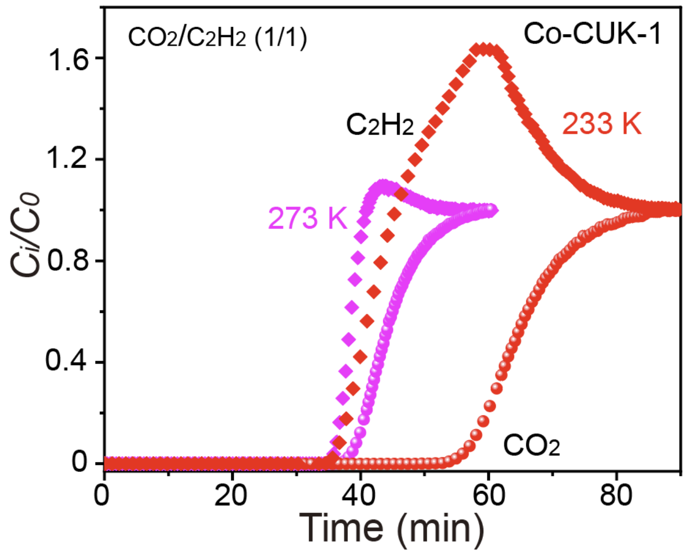

Supplementary Figure 20. Experimental breakthrough curves of CO_2/C_2H_2 (1/1) mixture on Co-CUK-1 at 273 and 233 K.

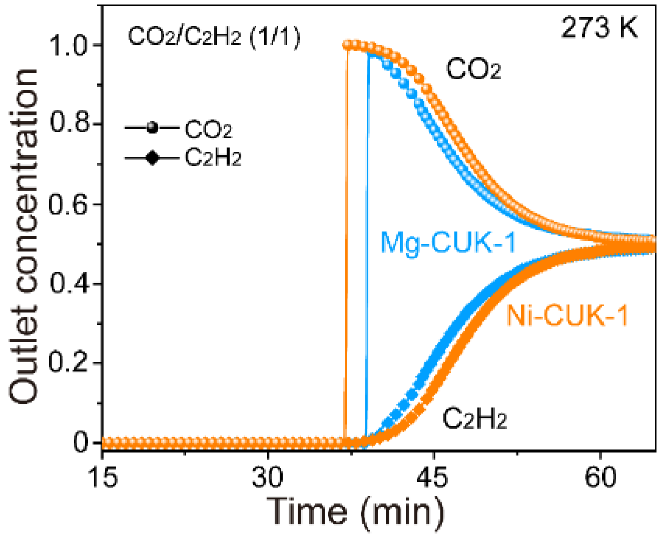

Supplementary Figure 21. Experimental breakthrough curves of CO_2/C_2H_2 (1/1) mixture on Mg-CUK-1 and Ni-CUK-1 at 273 K.

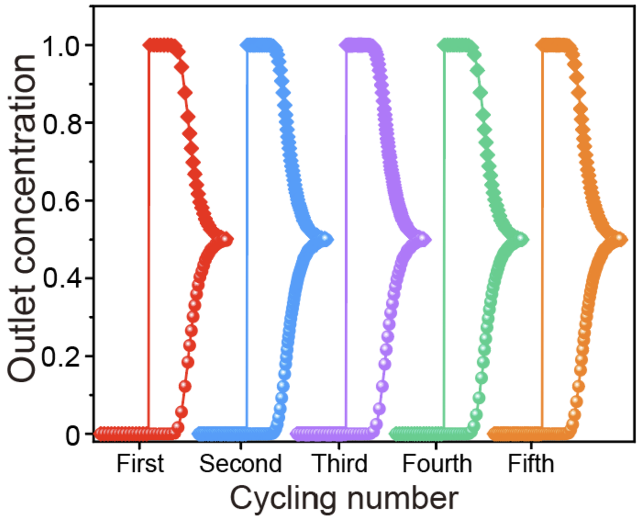

Supplementary Figure 22. Multiple-cycle breakthrough tests of Co-CUK-1 for CO_2/C_2H_2 (1/1) separation at 233 K.

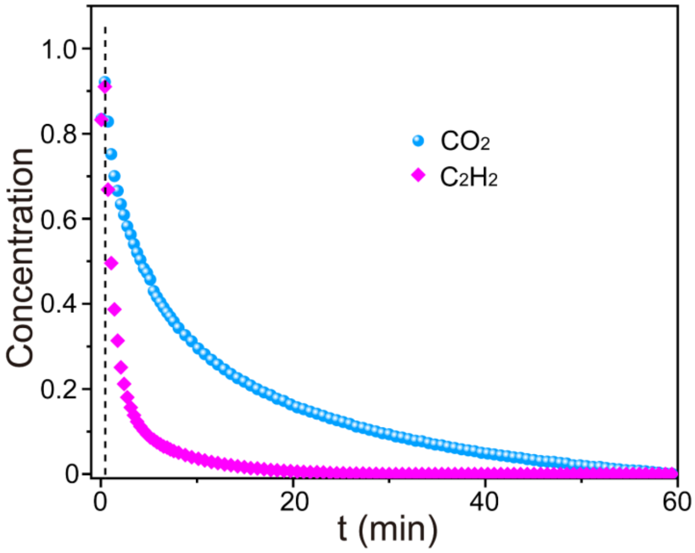

Supplementary Figure 23. Desorption curves of CO_2 and C_2H_2 from the fixed bed packed with Co-CUK-1 at 298 K with a He flow rate of 10 mL min^{-1}.

Supplementary Tables

Supplementary Table 1. The sorption uptakes and packing densities of CO_2 and C_2H_2 in CUK-1 materials at 1.0 bar and different temperatures.

	Pore volume (cm^3 g^{-1})	CO_2 uptake ($mmol$ g^{-1})		CO_2 packing density (cm^3 g^{-1})		C_2H_2 uptake ($mmol$ g^{-1})		C_2H_2 packing density (cm^3 g^{-1})	
Temperature (K)		233	298	233	298	233	298	233	298
Co-CUK-1	0.24	7.59	4.7	1.39	0.86	5.20	3.82	0.56	0.41
Ni-CUK-1	0.22	6.34	3.4	1.27	0.68	4.23	3.07	0.50	0.36
Mg-CUK-1	0.23	6.45	2.7	1.24	0.52	4.02	2.70	0.45	0.31

Supplementary Table 2. Calculated static binding energy of CO_2 and C_2H_2 in Co-CUK-1 at site I and site II.

	CO_2 at 233 K	CO_2 at 298 K	C_2H_2 at 233 K	C_2H_2 at 298 K
Site I (kJ mol^{-1})	43.47	26.59	33.34	38.34
Site II (kJ mol^{-1})	24.35	24.35	30.57	26.79

Supplementary Table 3. Calculated static binding energy of CO_2 and C_2H_2 in Ni-CUK-1 at site I and site II.

	CO_2 at 233 K	CO_2 at 298 K	C_2H_2 at 233 K	C_2H_2 at 298 K
Site I (kJ mol^{-1})	38.29	31.71	43.86	35.53
Site II (kJ mol^{-1})	29.42	21.12	22.42	29.74

Supplementary Table 4. Dual-site Langmuir-Freundlich parameter fits for CO_2 and C_2H_2 isotherms on Co-CUK-1 at 298 and 233 K.

	$q_{A,sat}$	b_A	v_A	$q_{B,sat}$	b_B	v_B
	mol kg^{-1}	Pa^{-vA}		mol kg^{-1}	Pa^{-vA}	
CO_2 at 233K	7.0405	0.05140	0.7682	4.5903	0.4096	1.2073
CO_2 at 298 K	3.44894	0.0096	1.16188	3.44894	0.0096	1.16188
C_2H_2 at 233 K	31.6951	0.009970	0.4057	3.2995	0.1364	0.7761
C_2H_2 at 298 K	1.4666	0.04293	1.68563	12.35615	0.01264	0.63236

Supplementary Table 5. Dual-site Langmuir-Freundlich parameter fits for CO_2 and C_2H_2 isotherms on Ni-CUK-1 at 298 and 233 K.

	$q_{A,sat}$	b_A	v_A	$q_{B,sat}$	b_B	v_B
	mol kg^{-1}	Pa^{-vA}		mol kg^{-1}	Pa^{-vA}	
CO_2 at 233K	3.6920	0.07229	0.9162	3.8759	0.9846	1. 6651
CO_2 at 298 K	2.40015	0.01109	1.15441	2.40015	0.01109	1.15441
C_2H_2 at 233 K	2.6822	0.03567	0.6952	2.6070	1.0808	1.9833
C_2H_2 at 298 K	1.8592	0.08025	1.25973	7.06312	0.01438	0.58319

Supplementary Table 6. Dual-site Langmuir-Freundlich parameter fits for CO_2 and C_2H_2 isotherms on Mg-CUK-1 at 298 and 233 K.

	$q_{A,sat}$	b_A	v_A	$q_{B,sat}$	b_B	v_B
	mol kg^{-1}	Pa^{-vA}		mol kg^{-1}	Pa^{-vA}	
CO_2 at 233K	3.67456	0.22954	1.6206	4.21514	0.11455	0.70461
CO_2 at 298 K	2.43155	0.00844	1.07196	2.43155	0.00844	1.07196
C_2H_2 at 233 K	3.55055	0.06477	1.18218	2.25899	0.05212	0.39
C_2H_2 at 298 K	1.66952	0.03333	1.02018	1.66952	0.03333	1.02018

Supplementary Table 7. Crystallographic data and details of guest-loaded Co-CUK-1.

Sample	CO_2@ Co-CUK-1	C_2H_2@ Co-CUK-1	CO_2@ Co-CUK-1	C_2H_2@ Co-CUK-1
Crystal system	Monoclinic	Monoclinic	Monoclinic	Monoclinic
Space group	*C*2/*c*	*C*2/*c*	*C*2/*c*	*C*2/*c*
Chemical formula	$Co_3(OH)_2(C_7H_3NO_4)_2(CO_2)_{3.47}$	$Co_3(OH)_2(C_7H_3NO_4)_2(C_2H_2)_{2.77}$	$Co_3(OH)_2(C_7H_3NO_4)_2(CO_2)_{2.07}$	$Co_3(OH)_2(C_7H_3NO_4)_2(C_2H_2)_{1.87}$
Temperature (K)	233	233	298	298
Light source	ESRF-ID22	ESRF-ID22	DLS-I11	DLS-I11
Wavelength (Å)	0.354267(4)	0.354267(4)	0.825829(1)	0.825829(1)
2θ range	1.5–25°	1.5–25°	3–50°	3–50°
a (Å)	18.12264(7)	17.9201(5)	17.8602(2)	17.9205(2)
b (Å)	12.70692(6)	13.0616(5)	13.1731(2)	13.0736(3)
c (Å)	10.96634(4)	10.9431(2)	10.83034(12)	10.85162(9)
β (°)	103.2495(3)	103.7394(12)	104.1601(5)	103.9468(9)
V (Å^3)	2458.138(18)	2488.10(13)	2470.68(6)	2467.42(7)
ρ (g cm^{-3})	1.875	1.637	1.700	1.588
R_{wp} (%)	9.50313	7.05498	14.58296	14.5431
R_p(%)	6.56087	4.97854	10.59034	10.7984
R_{Bragg}(%)	2.65654	1.69171	5.79452	4.77463
R_{exp} (%)	3.98694	1.52281	4.65654	5.93628
Gof χ^2	2.38356	4.63287	3.13172	2.44987

Supplementary Table 8. Crystallographic data and details of guest-loaded Ni-CUK-1.

Sample	CO_2@ Ni-CUK-1	C_2H_2@ Ni-CUK-1	CO_2@ Ni-CUK-1	C_2H_2@ Ni-CUK-1
Crystal system	Monoclinic	Monoclinic	Monoclinic	Monoclinic
Space group	*C*2/*c*	*C*2/*c*	*C*2/*c*	*C*2/*c*
Chemical formula	$Ni_3(OH)_2(C_7H_3NO_4)_2(CO_2)_{3.17}$	$Ni_3(OH)_2(C_7H_3NO_4)_2(C_2H_2)_{2.12}$	$Ni_3(OH)_2(C_7H_3NO_4)_2(CO_2)_{1.79}$	$Ni_3(OH)_2(C_7H_3NO_4)_2(C_2H_2)_{0.84}$
Temperature (K)	233	233	298	298
Light source	ESRF-ID22	ESRF-ID22	DLS-I11	DLS-I11
Wavelength (Å)	0.354267(4)	0.354267(4)	0.825829(1)	0.825829(1)
2θ range	1.5–25°	1.5–25°	3–50°	3–50°
a (Å)	17.90429(13)	17.7715(3)	17.48128(10)	17.8889(3)
b (Å)	12.70214(10)	12.9344(2)	13.39860(12)	12.7231(2)
c (Å)	10.82143(6)	10.81466(12)	10.68687(5)	10.81868(15)
β (°)	103.2504(6)	103.6442(12)	104.8004(5)	103.2905(5)
V (Å^3)	2395.52(3)	2415.75(6)	2420.08(3)	2396.41(6)
ρ (g cm^{-3})	1.897	1.634	1.701	1.555
R_{wp} (%)	8.17760	11.25646	14.66610	11.25330
R_p(%)	5.98970	7.57953	10.30109	7.84831
R_{Bragg}(%)	2.69002	4.12050	6.87370	2.70892
R_{exp} (%)	3.24546	1.38777	5.11772	4.82745
Gof χ^2	2.51971	8.11120	2.86575	2.33111

Supplementary Table 9. Summary of the adsorption uptakes and selectivities for CO_2-selective materials.

Materials	CO_2 uptake ($cm^3\ g^{-1}$)	C_2H_2 uptake ($cm^3\ g^{-1}$)	Temperature (K)	IAST selectivity (50/50)	*Ref.*
Co-CUK-1	170	119	233	9.5	*This work*
Mg-CUK-1	144	89	233	12.1	
Cu-F-pymo	26.6	2.3	298	>10^5	[5]
PMOF-1	53.3	7.5	273	694	[6]
Ce(IV)-MIL-140-4F	110.3	41.5	298	44	[7]
MUF-16	47.8	4.0	298	510	[1]
CD-MOF-2	59.6	45.5	298	12.8	[8]
Tm-OH-bdc	130.6	47	298	18.2	[9]
SIFSIX-3-Ni	60.5	73.9	298	7.5	[10]
PCP-NH_2-ipa	72	43.7	298	6.4	[11]
[Mn(bdc)(dpe)]	46.8	7.3	273	8.8	[12]

References

1 Qazvini, O. T., Babarao, R., Telfer, S. G. Selective capture of carbon dioxide from hydrocarbons using a metal-organic framework. *Nat. Commun.* **12**, 197 (2021).

2 Walton, K. S., Sholl, D. S. Predicting multicomponent adsorption: 50 Years of the ideal adsorbed solution theory. *AIChE J.* **61**, 2757-2762 (2015).

3 Myers, A. L., Prausnitz, J. M. Thermodynamics of mixed gas adsorption. *AIChE J.* **11**, 121-127 (1965).

4 Zhang, Z., Yang, Q., Cui, X., Yang, L., Bao, Z., Ren, Q., Xing, H. Sorting of C_4 olefins with interpenetrated hybrid ultramicroporous materials by combining molecular recognition and size-sieving. *Angew. Chem. Int. Ed.* **56**, 16282-16287 (2017).

5 Shi, Y., Xie, Y., Cui, H., Ye, Y., Wu, H., Zhou, W., Arman, H., Lin, R. B., Chen, B. Highly selective adsorption of carbon Dioxide over acetylene in an ultramicroporous metal-organic framework. *Adv. Mater.* **33**, 2105880 (2021).

6 Cai, L. Z., Yao, Z. Z., Lin, S. J., Wang, M. S., Guo, G. C. Photoinduced electron-transfer (PIET) strategy for selective adsorption of CO_2 over C_2H_2 in a MOF. *Angew. Chem. Int. Ed.* **60**, 18223-18230 (2021).

7 Zhang, Z., Peh, S. B., Krishna, R., Kang, C., Chai, K., Wang, Y., Shi, D., Zhao, D. Optimal pore chemistry in an ultramicroporous metal-organic framework for benchmark inverse CO_2/C_2H_2 separation. *Angew. Chem. Int. Ed.* **60**, 17198-17204 (2021).

8 Li, L., Wang, J., Zhang, Z., Yang, Q., Yang, Y., Su, B., Bao, Z., Ren, Q. Inverse adsorption separation of CO_2/C_2H_2 mixture in cyclodextrin-based metal-organic frameworks. *ACS Appl. Mater. Interfaces* **11**, 2543-2550 (2019).

9 Ma, D., Li, Z., Zhu, J., Zhou, Y., Chen, L., Mai, X., Liufu, M., Wu, Y., Li, Y. Inverse and highly selective separation of CO_2/C_2H_2 on a thulium–organic framework. *J. Mater. Chem. A* **8**, 11933-11937 (2020).

10 Chen, K. J., Scott, H. S., Madden, D. G., Pham, T., Kumar, A., Bajpai, A., Lusi, M., Forrest, K. A., Space, B., Perry, J. J., *et al.* Benchmark C_2H_2/CO_2 and CO_2/C_2H_2 separation by two closely related hybrid ultramicroporous materials. *Chem* **1**, 753-765 (2016).

11 Gu, Y., Zheng, J. J., Otake, K. I., Shivanna, M., Sakaki, S., Yoshino, H., Ohba, M., Kawaguchi, S., Wang, Y., Li, F., *et al.* Host-guest interaction modulation in porous coordination polymers for inverse selective CO_2/C_2H_2 separation. *Angew. Chem. Int. Ed.* **60**, 11688-11694 (2021).

12 Foo, M. L., Matsuda, R., Hijikata, Y., Krishna, R., Sato, H., Horike, S., Hori, A., Duan, J., Sato, Y., Kubota, Y., *et al.* An adsorbate discriminatory gate effect in a flexible porous coordination polymer for selective adsorption of CO_2 over C_2H_2. *J. Am. Chem. Soc.* **138**, 3022-3030 (2016).
